# Genomic and Transcriptomic Analysis of Pea (*Pisum sativum* L.) Breeding Line ‘Triumph’ with High Symbiotic Responsivity

**DOI:** 10.3390/plants13010078

**Published:** 2023-12-26

**Authors:** Evgeny A. Zorin, Anton S. Sulima, Aleksandr I. Zhernakov, Daria O. Kuzmina, Valeria A. Rakova, Marina S. Kliukova, Daria A. Romanyuk, Olga A. Kulaeva, Gulnar A. Akhtemova, Oksana Y. Shtark, Igor A. Tikhonovich, Vladimir A. Zhukov

**Affiliations:** 1 All-Russia Research Institute for Agricultural Microbiology (ARRIAM), Pushkin, St. Petersburg 196608, Russia; ezorin@arriam.ru (E.A.Z.); asulima@arriam.ru (A.S.S.); azhernakov@arriam.ru (A.I.Z.); kuzminad16@gmail.com (D.O.K.); mkliukova@arriam.ru (M.S.K.); d.romanyuk@arriam.ru (D.A.R.); okulaeva@arriam.ru (O.A.K.); gakhtemova@arriam.ru (G.A.A.); oshtark@arriam.ru (O.Y.S.); arriam2008@yandex.ru (I.A.T.); 2 Center of Genetics and Life Sciences, Sirius University of Science and Technology, Sirius 354340, Russia; rakova.va@learn.siriusuniversity.ru

**Keywords:** plant–microbe symbiosis, legumes, *Pisum sativum*, symbiotic responsivity, genomics, transcriptomics, differential gene expression, molecular breeding

## Abstract

Pea (*Pisum sativum* L.), like most legumes, forms mutualistic symbioses with nodule bacteria and arbuscular mycorrhizal (AM) fungi. The positive effect of inoculation is partially determined by the plant genotype; thus, pea varieties with high and low symbiotic responsivity have been described, but the molecular genetic basis of this trait remains unknown. Here, we compare the symbiotically responsive breeding line ‘Triumph’ of grain pea with its parental cultivars ‘Vendevil’ (a donor of high symbiotic responsivity) and ‘Classic’ (a donor of agriculturally valuable traits) using genome and transcriptome sequencing. We show that ‘Triumph’ inherited one-fourth of its genome from ‘Vendevil’, including the genes related to AM and nodule formation, and reveal that under combined inoculation with nodule bacteria and AM fungi, ‘Triumph’ and ‘Vendevil’, in contrast to ‘Classic’, demonstrate similar up-regulation of the genes related to solute transport, hormonal regulation and flavonoid biosynthesis in their roots. We also identify the gene *PsGLP2*, whose expression pattern distinguishing ‘Triumph’ and ‘Vendevil’ from ‘Classic’ correlates with difference within the promoter region sequence, making it a promising marker for the symbiotic responsivity trait. The results of this study may be helpful for future molecular breeding programs aimed at creation of symbiotically responsive cultivars of pea.

## 1. Introduction

Legumes (family *Fabaceae*) are a specific group of plants capable of forming two types of mutualistic symbioses: root nodules (RN) with nitrogen-fixing bacteria (rhizobia) and arbuscular mycorrhiza (AM) with the fungi of the order *Glomeromycota* [1,2,3]. These symbioses are beneficial for both the host plant and the microsymbionts, as well as for the environment. Indeed, RN and AM improve mineral nutrition by supplying plants with macroelements—nitrogen and phosphorus, respectively—thus increasing the plants’ fitness, yield and stress resilience. In turn, rhizobia and AM fungi receive carbon compounds produced by the plant, mainly in the form of succinate and malate for rhizobia and in the form of carbohydrates and fatty acids for AM fungi. Lastly, the symbioses improve the soil structure and fertility: the amount of N_2_ fixation by legumes is estimated to be up to 300 kg N ha^−1^ year^−1^, depending on the legume species and the method of assessment [4,5,6], and AM stabilizes soil macro-aggregation, and, thus, protects the soil from erosion [7]. These advantages associated with a symbiotic lifestyle make legumes ideal crops for use in the modern approach of sustainable agriculture [8].

The molecular mechanisms underlying the formation and functioning of RN and AM symbioses have been well studied, especially in the model legumes such as *Medicago truncatula* Gaertn. (barrel medik), *Lotus japonicus* (Regel.) K. Larsen and *Glycine max* (L.) Merr. (soybean) [1,9]. There is, however, one aspect of symbiosis where our understanding is still lacking: symbiotic efficiency and its genetic control. Indeed, strains of rhizobia differ in their nitrogen fixation capabilities [10,11]; moreover, some rhizobial strains are incompatible with particular varieties of legume plants due to the features of both plant and microbe genotypes. Similarly, some isolates of AM fungi have a less-beneficial effect on plants than the others (the genetic basis of this trait is unknown), and plant species (and, possibly, even plants within the same species) differ in their mycorrhizal dependency (which is determined as the degree of plant growth change associated with arbuscular mycorrhizal colonization) [12]. One can generalize that the plant’s genome apparently affects the effectiveness of the formed symbioses, but the genetic determinants responsible for this trait are still to be elucidated.

Pea (*Pisum sativum* L.) is an important legume crop worldwide (FAOSTAT, 2021) and a promising candidate for use in sustainable agriculture. During the last decade, significant advances in the area of genetics and genomics of pea have been made, including the development of useful modern tools such as reference genomes, pan-genomes, transcriptomic atlases and several sets of molecular markers for marker-assisted breeding (reviewed in [13]). Currently, pea breeding is mostly aimed at improvement of pathogen resistance and stress tolerance, with less attention to symbiosis-related traits [14,15]. However, several years ago, it was proposed to consider the increase in seed biomass due to the complex inoculation with rhizobia and AM fungi as a trait for legume breeding [16]. This integral trait was named Efficiency of Interactions with Beneficial Soil Microorganisms (EIBSM), or symbiotic responsivity [8,16]. In accordance with this proposal, pea genotypes with high symbiotic responsivity have been identified in the germplasm collection of the N.I. Vavilov All-Russian Institute of Plant Genetic Resources (VIR) [17,18] and subsequently involved in breeding programs. As a result of backcrossing of cultivar ‘Classic’ (a donor of plant architectonics) on cultivar ‘Vendevil’ (=k-8274 in the VIR collection, a donor of symbiotic responsivity), the pea breeding line with increased EIBSM named ‘Triumph’ has been developed [19]. Symbiotic responsivity of the breeding line ‘Triumph’ has been successfully proven in three-year field trials under inoculation with nodule bacteria and AM fungi [20].

Since the EIBSM trait could be of high interest for pea breeders, we aimed to characterize the molecular genetic basis of this trait in order to pave the way for further molecular breeding programs in pea. To this end, we sequenced, analyzed and compared the genomes and transcriptomes of ‘Triumph’ and its parental cultivars, ‘Classic’ and ‘Vendevil’. Our findings demonstrate that: (i) ‘Triumph’ inherited 22.5% of its genome from ‘Vendevil’, including the genes annotated as related to AM and nodule formation, (ii) under the combined inoculation with nodule bacteria and AM fungi ‘Triumph’ and ‘Vendevil’ similarly show up-regulation of genes involved in nodules and AM development, which are related to solute transport, hormone regulation and flavonoid biosynthesis, and (iii) the expression of one of such genes, namely, the gene encoding a germin-like protein, which we named *PsGLP2*, is associated with differences in its promoter region, that makes it a promising marker of the symbiotic responsivity.

## 2. Results

### 2.1. Genome Sequencing of the Breeding Line ‘Triumph’ and Its Parental Cultivars

In order to characterize the genome composition of ‘Triumph’, we sequenced its nuclear genome along with genomes of parental cultivars ‘Classic’ and ‘Vendevil’, and analyzed it using the reference genome of pea cultivar ‘Frisson’ obtained earlier in our workgroup [21]. We sought for the genes inherited by ‘Triumph’ from ‘Vendevil’, since these genes could be responsible for the high symbiotic responsivity.

#### 2.1.1. Sequencing and Data Processing

The genomes of all three genotypes were sequenced on Illumina NovaSeq 6000 in Sirius University of Science and Technology (Sirius, Russia), and a total of 360 Gb of raw data was obtained. After the low-quality reads were filtered out, the high-quality paired-end reads were mapped to the reference pea genome of cv. ‘Frisson’ (NCBI accession number: JANEYU000000000 [21]). On average, more than 90% of the reads were unambiguously mapped to the reference (Supplementary Table S1).

By comparing the three genomes to the reference, a total of 19,375,034 variations, including single-nucleotide variants (SNVs) and insertions–deletions (indels), were identified. As expected, most of the variations are located in intergenic regions. In addition, many variations have fallen on upstream and downstream regions, which may correspond to the putative promoter and the enhancer/silencer regions. A considerable number of variations also correspond to ORFs, affecting both introns and exons (Supplementary Figure S1).

#### 2.1.2. Portion of the ‘Triumph’s’ Genome Inherited from cv. ‘Vendevil’

In order to determine which genes ‘Triumph’ inherited from ‘Vendevil’, we decided to consider the gene structure in a broad sense, that is, including exons, introns, as well as 5′ and 3′ UTRs and putative promoter sites (i.e., 1000 bp in the upstream region). Therefore, we analyzed SNVs and indels located in any of the structural parts of the gene mentioned above and leading to both synonymous and non-synonymous substitutions. At the same time, variations were considered only in protein-coding genes, and all mobile genetic elements were removed from the analysis.

The number of SNVs shared by ‘Vendevil’ and ‘Triumph’ and different from ‘Classic’ is 21,467 in 7582 genes, of which 13,081 substitutions fall into putative regulatory regions located upstream to the genes, 2138 fall in the 5′ and 3′ UTR regions, introns account for 4266 SNVs, and 989 SNVs fall into exons. Among the substitutions found in exons, 308 are synonymous and 581 are non-synonymous.

In addition, we searched for indels ranging in size from 1 to 99 bp and found 785,511 in all three genotypes combined, among which 398,493 were insertions and 387,018—deletions (compared to the ‘Frisson’ genome). Of those, in total, 41,250 indels were inherited by ‘Triumph’ from ‘Vendevil’.

Since a significant part of the pea genome consists of mobile elements of various classes with a high frequency of mutations, only genes encoding already known proteins and not related to mobile elements were selected for further work. As a result of filtering, 2856 protein-coding genes with UTRs and putative regulatory sequences containing 15,177 SNVs remained (Supplementary Figure S2). After removal of mobile genetic elements and genes encoding unknown proteins, 6971 indels fall into 1150 known protein-coding genes (including 5’ and 3’ UTRs and 1000 bp upstream the ORF, see above). At the same time, only 101 indels are in the ORFs of 66 genes (Supplementary Figure S2).

**Figure 1:**
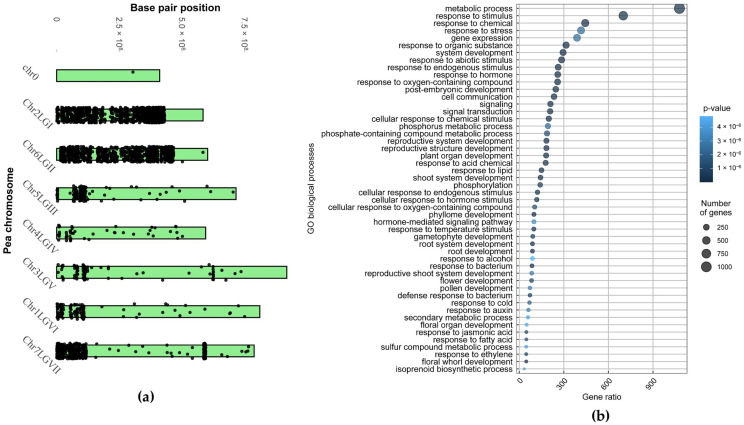
Genomic and functional distribution of genes that ‘Triumph’ inherited from ‘Vendevil’. (**a**) Distribution of genes inherited by ‘Triumph’ from ‘Vendevil’ by chromosomes and linkage groups. Genes on chromosomes are applied as dots. Chr—chromosome; LG—linkage group. (**b**) Gene enrichment analysis of genes inherited by ‘Triumph’ from ‘Vendevil’. Point size is proportional to the number of genes in a particular group.

Interestingly, the genes are not distributed evenly, and ‘Triumph’ apparently inherited a large portion of the second and sixth chromosomes from ‘Vendevil’, while major regions of other chromosomes are inherited from ‘Classic’ (Figure 1a and Table 1). In total, out of 35,723 genes annotated in the genome of ‘Frisson’, ‘Triumph’ inherited 2586 genes with SNVs and 1150 genes with indels from ‘Vendevil’, 12,868 from ‘Classic’, and other genes were non-polymorphic. Based on these numbers, we surmised that the percentage of the genome inherited by ‘Triumph’ from ‘Vendevil’ was 22.5% (3736/(3736 + 12,868) × 100% = 22.5%).

To gain an overview of genes that were inherited by ‘Triumph’ from ‘Vendevil’, the Gene Ontology (GO) enrichment analysis was performed. As a result, it was shown that these genes relate primarily to such common biological processes as ‘metabolic process’, ‘response to stimulus’, ‘response to stress’, ‘response to chemical’, ‘gene expression’, ‘response to oxygen-containing compounds’, ‘response to organic substance’, ‘response to hormone’ and ‘response to endogenous stimulus’ (Figure 1b). However, genes from categories such as ‘phosphorus metabolic process’, ‘response to lipid’, ‘defense response to bacterium’, ‘response to bacterium’, ‘isoprenoid biosynthetic process’ may be responsible for the manifestation of phenotypic traits inherited by ‘Triumph’ from ‘Vendevil’. In addition, the genes involved in ‘response to symbiotic bacterium’ and ‘arbuscular mycorrhizal association’ were identified by analyzing the dataset for genes with indels in ORF separately, as these genes are few in number and are concealed in a large dataset (Supplementary Figure S3).

Regarding the symbiotic genes, i.e., the genes with known roles in RN or AM symbiosis, only the genes *NRAMP1* (nonsynonymous SNV in ORF) encoding ferrous ion transporter [22], *ERF1* (deletion in 3′ UTR) encoding transcription factor that positively affects nodulation [23], *PHYB* (deletion in 5′ UTR) encoding phytochrome B [24], *FLOT4* (deletion in 5′ UTR) encoding flotillin [25], and *PUB1* (conservative inframe deletion, i.e., the codon has been deleted) encoding E3 ubiquitin ligase [26] came from ‘Vendevil’, and therefore can be considered as possible factors of symbiotic responsivity, given that the efficiency of ion transport through symbiotic membranes may affect the efficiency of nitrogen fixation, and the other genes are known for their important function in RN and AM symbioses [9].

### 2.2. Transcriptomic Analysis of Responses of ‘Triumph’, ‘Vendevil’ and ‘Classic’ to Inoculation with Rhizobia and AM Fungi

In order to study the realization of genetic information in ‘Triumph’, we assessed the gene expression in 4-week-old shoots and roots of the three genotypes under simultaneous inoculation with nodule bacteria and AM fungi in a pot experiment. At the same time, growth parameters were estimated in plants aged approximately 3 months after planting.

#### 2.2.1. The Effect of Combined Inoculation with Nodule Bacteria and AM Fungi on Growth Parameters of the Studied Pea Genotypes

**Figure 2:**
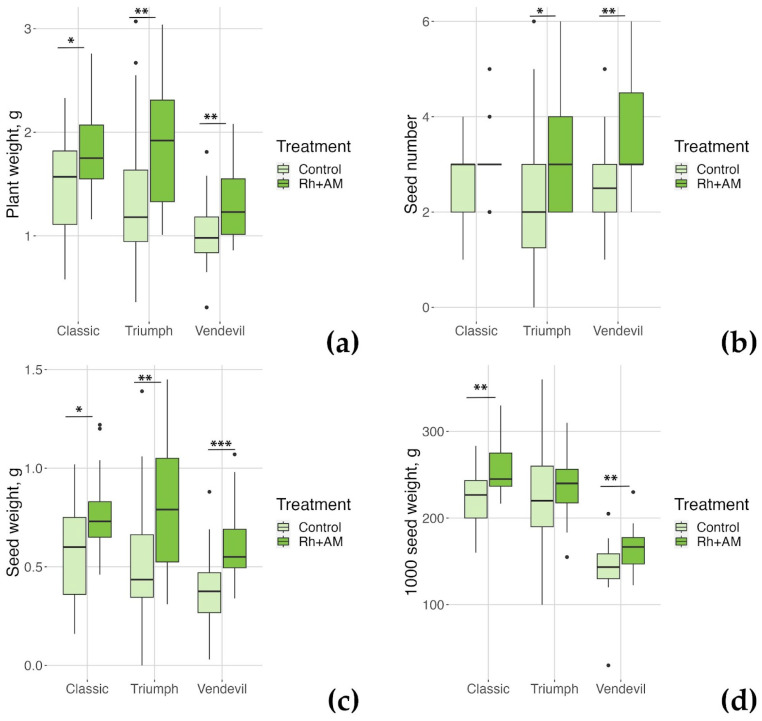
Boxplots demonstrating physiological parameters of genotypes measured after vegetation. (**a**) plant weight; (**b**) seed number; (**c**) seed weight; (**d**) 1000 seed weight. *—*p*-value is < 0.05, **—*p*-value is < 0.01, ***—*p*-value is < 0.001.

Four weeks after planting and inoculation, the effect of inoculation on the measured growth parameters was slight: the fresh shoot weight of ‘Vendevil’ only increased due to inoculation, and for the fresh root weight of ‘Classic’ a tendency for decrease was noted (Supplementary Figures S4–S6). At the same time, the fresh shoot weight of ‘Vendevil’ was significantly less than that of ‘Triumph’ and ‘Classic’ in both control conditions and under inoculation (Supplementary Figures S4–S6). The number of nodules did not differ significantly between genotypes in inoculated pots, possibly due to the small number of plants processed (Figure 2); we also detected few nodules on the roots of plants in control pots (possibly, the pots were contaminated during watering). At the end of the experiment, the effect of inoculation on plant and seed weight was statistically significant for all genotypes; however, the seed number increased due to combined inoculation for ‘Vendevil’ and ‘Triumph’ only. Moreover, the seed weight increased approximately 50% for ‘Vendevil’ and ‘Triumph’, and only 30% for ‘Classic’; the percentage of increase in dry weight of plants (sum of root and shoot weights measured separately) was approx. 30% for ‘Vendevil’ and ‘Triumph’, and approx. 20% for ‘Classic’. Thus, the EIBSM trait manifested itself in our experiment, and we demonstrated that the response to inoculation of ‘Triumph’ resembles the one of ‘Vendevil’ and differs from that of ‘Classic’, thus suggesting the inheritance of the symbiotic responsivity trait from ‘Vendevil’.

#### 2.2.2. Transcriptomic Analysis of the Response to Inoculation

To analyze the gene expression patterns in the studied genotypes, a differential gene expression analysis was carried out. To carry this out, high-quality sequencing reads were mapped to the reference pea genome of cv. ‘Frisson’, and a gene expression matrix was constructed. Of the 23,838 genes with a non-zero expression value, 204, 195 and 245 genes were differentially expressed (up- and down-regulated) in response to inoculation in the roots of ‘Classic’, ‘Triumph’ and ‘Vendevil’, respectively.

Principal component analysis revealed that inoculation has a greater influence on the gene’s expression than the plant genotype (Supplementary Figure S7). Accordingly, all three genotypes have 44 common genes up-regulated in response to inoculation (Figure 2a and Supplementary Table S2). Among them, there are genes encoding mycorrhiza-specific transporters: phosphate transporter PT8 (homolog of *MTR_5g068140*) [27], ammonium transporter AMT2;5 (homolog of *MTR_1g036410*) [28], and ABC transporter STR2 (homolog of *MTR_5g030910*) [29,30]. Also, there are genes with function associated with RN or AM symbioses, encoding legume lectin proteins (homologs of *M. truncatula MTR_5g031160*, *MTR_5g031090*, *MTR_8g068040*), Nodulin-26-like intrinsic protein (NIP) (homolog of *MTR_8g087710*) [31], nitrate transporters (NRT1) (homologs of *MTR_7g098040* and *MTR_8g087810*) [32], amino acid transporter (homolog of *MTR_2g101920*), lipid transfer protein (homolog of *MTR_4g076150*), as well as indole-3-acetic acid-amido synthetase (homolog of *MTR_5g016320*) and a GRAS family transcription factor (homolog of *MTR_2g089100*). Homologs of some genes from this group, such as *MTR_2g089100*, *MTR_4g081190*, *MTR_8g087710*, *MTR_5g075400*, *MTR_5g045470* and *MTR_5g031160*, are also present in the list of arbuscular mycorrhiza-induced genes inferred from a meta-analysis of transcriptomic data and called biosignatures of AM colonization [33].

**Figure 3:**
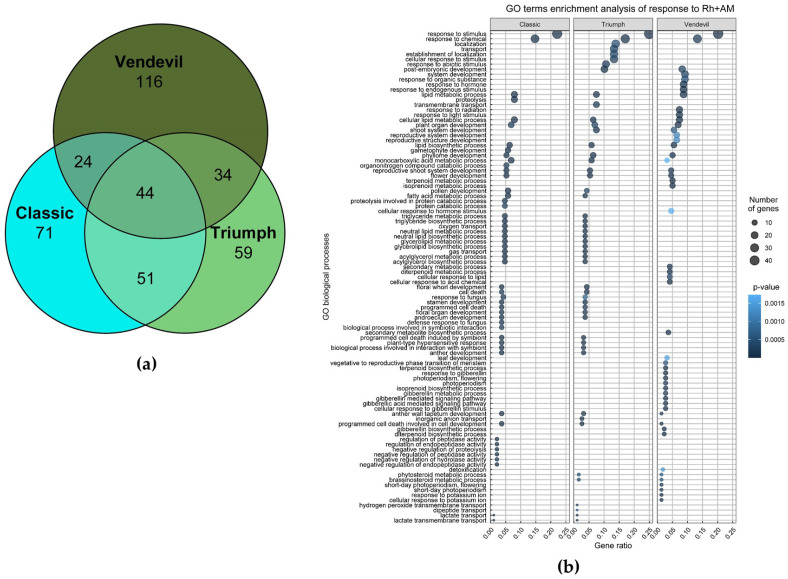
Comparison of up-regulated genes in three genotypes in response to inoculation. (**a**) Venn diagram showing similarities and differences in up-regulated genes in three genotypes in response to inoculation with rhizobia and mycorrhizal fungi. (**b**) Gene ontology biological process analysis of up-regulated genes in response to inoculation of rhizobia and mycorrhizal fungi.

Among the studied genotypes, ‘Vendevil’ demonstrated the most intense response to inoculation (218 up-regulated DEGs), which is consistent with the idea of its higher symbiotic responsivity. The expression profiles of the roots of ‘Triumph’ and ‘Classic’ were more similar to each other than to ‘Vendevil’ (51 common up-regulated DEGs) (Figure 3a); the GO enrichment analysis also showed that the response of ‘Triumph’ and ‘Classic’ to a combined inoculation is quite similar (Figure 3b). However, the common terms of GO biological processes shared by ‘Triumph’ and ‘Vendevil’ (and not present in ‘Classic’) were also found, and those were related to phytosteroid metabolic processes. The corresponding genes are listed in Table 2. These genes are related to hormonal regulation, in particular, the homologous pea genes of the *MTR_7g102460* are involved in cytokinin catabolism, while the pea homolog of the *MTR_5g082520* gene participates in the biosynthesis of brassinosteroids.

The studied genotypes also demonstrated a down-regulation of a number of genes due to inoculation, but this response was weak and specific for each genotype, so no common differentially down-regulated genes were identified (Supplementary Table S3).

**Figure 4:**
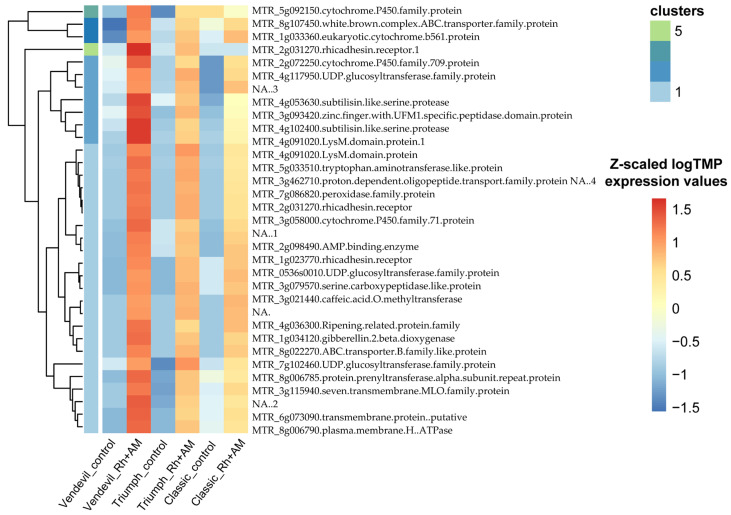
Heatmap demonstrating the expression profile of common ‘Triumph’ and ‘Vendevil’ DEGs in response to inoculation of roots. Rh + AM—inoculation by rhizobia and AM fungi. The color scale indicates normalized expression values (using TPM method) for each gene after Z-transformation.

Next, we analyzed the set of genes that were similarly up-regulated in the roots of ‘Triumph’ and ‘Vendevil’ and did not significantly change the expression level in the roots of ‘Classic’ under inoculation. This group consisted of 34 genes, most of which increased the expression level in response to double inoculation in all three genotypes, but the change in expression was significant only for ‘Triumph’ and ‘Vendevil’ but not for ‘Classic’ (Figure 4, Supplementary Table S4). Homologs of some of these genes are mycorrhiza-induced genes (and are present in the AM biosignature set [33]: *MTR_8g022270*, *MTR_3g115940*, *MTR_4g102400*, *MTR_3g058000*, and *MTR_5g092150*). The AM-specific gene *STR* (homolog of *MTR_8g107450*) was also significantly up-regulated only in the roots of ‘Triumph’ and ‘Vendevil’. Additionally to the mycorrhiza-induced genes, the genes related to biosynthetic and metabolic pathways of biologically active molecules such as flavonoids, gibberellic acid and auxin (Table 3) were found in this group.

In contrast to the roots, the response to inoculation in the shoots was dismissively weak and specific for each genotype.

#### 2.2.3. Allele-Specific Gene Expression in Shoots and Roots of ‘Triumph’

To analyze the allele-specific expression of genes inherited by ‘Triumph’ from ‘Vendevil’, we compared the gene expression separately in roots and in shoots of ‘Triumph’ and ‘Vendevil’ vs. ‘Classic’ in control conditions and under combined inoculation. As a result, we selected 39 genes with similar expression patterns in ‘Vendevil’ and ‘Triumph’ (Supplementary Figure S8 and Table S5). The intersection of this gene list with the table of variations (SNVs + indels) allowed us to identify genes that have an allele-specific expression pattern in ‘Triumph’ and ‘Vendevil’ (Supplementary Table S5, highlighted in bold).

**Figure 5:**
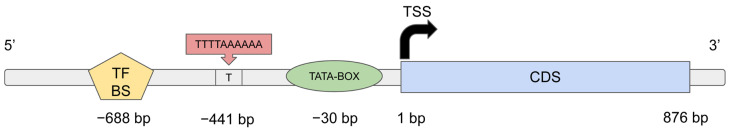
Schematic structure of the gene encoding germin-like protein with the promoter region carrying the insertion. CDS—coding sequence; TSS—transcription start site; TF BS—transcription factor binding site.

In control conditions, two genes were identified that encoded disease resistance response protein Pi49 (*M. truncatula* homolog ID—*MTR_2g035150*) and cucumisin protein (no *M. truncatula* homologs were identified; the closest is *Vicia villosa* homolog ID—*LOC131640492*) (Supplementary Table S5). Under inoculation, two genes demonstrated an allele-specific expression pattern. One of them encodes the 12-oxophytodienoate reductase 1-like protein (ORP; *M. truncatula* homolog ID—*MTR_5g006740*), which participates in the jasmonic acid synthesis [34]. The second gene encodes a germin-like protein (GLP). Phylogenetic analysis showed that it most closely resembles *MTR_2g023770* from *M. truncatula* with the putative function of the rhicadhesin receptor (Supplementary Figure S10). We named it *PsGLP2*, for *Pisum sativum Germin-like protein 2*. The gene has identical sequence in the protein-coding part in all three genotypes, but contains a shared 9 bp insertion for ‘Triumph’ and ‘Vendevil’ in the putative regulatory region upstream of CDS (Figure 5). Accordingly, this is the only gene whose expression level increases significantly upon inoculation in ‘Triumph’ and ‘Vendevil’ but does not change in ‘Classic’ (log10 Fold Change values in ‘Vendevil’ and ‘Triumph’—4.56 (adjusted *p*-value—0.0008) and 5.08 (adjusted *p*-value—0.0169), respectively (Figure 4). This finding indicates that the induction of expression of this gene might be the genetic base of the symbiotic responsivity in pea.

#### 2.2.4. qRT-PCR Validation of Transcriptomic Data

In addition, we performed qRT-PCR to confirm the results obtained by transcriptome analysis. In total, four genes involved in flavonoid biosynthesis and gibberellin metabolism, together with the marker gene *PsGLP2*, were selected for qRT-PCR validation. The results of qRT-PCR for these genes matched the transcriptomic data with correlation coefficient ranged from 0.82 to 0.97, confirming the reliability of the transcriptome analysis (Supplementary Figure S11).

## 3. Discussion

The mutualistic symbiosis with rhizobia has long been known to have a beneficial effect on legumes, allowing them to grow in nitrogen deficiency and increasing their fitness and yield. Since there are many crops among legumes, the ability to form an effective symbiosis with bacteria should be considered as a valuable trait for breeding [35]. In turn, since arbuscular mycorrhizal fungi are present in virtually all cultivated soils, the ability to form an effective AM can also be a subject for legume crops’ breeding. In order to simplify and formalize the breeding process, we previously proposed a quantitative metric for the effectiveness of symbiosis (EIBSM, for Effectiveness of Interaction with Beneficial Soil Microorganisms), or, in other words, the responsivity of the plant to the symbiotic microorganisms [8,16]. An example of the practical application of this metric is the pea breeding line ‘Triumph’, which demonstrates increased responsivity to inoculation with nodule bacteria and AM fungi. 

The breeding line ‘Triumph’ was created as a result of five backcrosses of the cv. ‘Vendevil’ (=k-8274 in the VIR pea genetic collection) with cv. ‘Classic’ (The Netherlands) followed by four generations of selfing with simultaneous selection for high yield under inoculation with nodule bacteria and AM fungi [19]. The idea of this breeding program was to combine high EIBSM with agriculturally important traits such as the semi-leafless phenotype (inherent to ‘Classic’ due to the mutation in the *afila* (*af*) gene) and high seed productivity [36]. The resulting breeding line ‘Triumph’ has proved its symbiotic responsivity in three-year field trials [20], but no further characterization of this line was performed, except for the study of Kuzmicheva et al. (2014), in which ‘Triumph’ was shown to excrete high amounts of pyruvic and succinic acids in the root exudates, similar to its parental cultivars ‘Vendevil’, while ‘Classic’ excreted low amounts of those organic acids [37]. 

In the present work, a similar experimental design was used, i.e., ‘Triumph’ was compared to its parental cultivars ‘Vendevil’ and ‘Classic’ under simultaneous inoculation with nodule bacteria and AM fungi. In our inoculation experiment ‘Triumph’ and its parental cultivar ‘Vendevil’, the donor of EIBSM, responded to inoculation more readily than the second parental cultivar ‘Classic’, the donor of plant architectonic, even despite the fact of rhizobial contamination in control pots. Interestingly, ‘Classic’ demonstrated a tendency to form fewer roots with more nodules than ‘Triumph’ and ‘Vendevil’; although, this difference was not statistically significant. This fact, however, coincides with the observed down-regulation of several nodule-specific genes such as those encoding leghemoglobin and lectins in the roots of ‘Triumph’ and ‘Vendevil’ as compared to ‘Classic’ (Supplementary Table S3). Further experiments are required to establish the connection between the regulation of the nodule number from the plant side and the symbiotic effectiveness in pea.

The results of our analysis point towards the important role of plant hormones in determining the effectiveness of symbiosis with nodule bacteria and AM fungi. Among the similarly regulated genes in roots of ‘Triumph’ and ‘Vendevil’, we found genes presumably involved in the biosynthesis pathways of ABA, cytokinins, gibberellins and brassinosteroids. Unfortunately, the annotation data currently available in the databases do not allow us to draw a clear conclusion about differences in biological processes; for example, according to BLAST, the *evm.TU.scaffold_258.357* gene up-regulated in ‘Triumph’ and ‘Classic’ (Table 3) is encoding a flavonol synthase, but its closest homolog in *M. truncatula* is annotated as 2-oxoglutarate-dependent dioxygenase, an enzyme participating in gibberellin synthesis [38,39,40]. Lange and Lange, 2020, say that such cases are not uncommon, and homologs of GA 7-oxidase (GA7ox) are often called flavonol synthases [39]. Thus, at the moment we cannot be certain that the gibberellins affect the EIBSM. However, gibberellins are known to block the infection during RN symbiosis while stimulating the growth and development of the existing nodules [41]; indeed, in our experiment, ‘Classic’ formed many small nodules, while in ‘Triumph’ and ‘Vendevil’, nodules were less numerous. It is also worth mentioning that succinic acid, which is abundant in root exudates of ‘Triumph’ and ‘Vendevil’ [37], is a by-product of the reaction catalyzed by 2-oxoglutarate-dependent dioxygenase (gibberellin 2-beta-dioxygenase), which might be encoded by *evm.TU.scaffold_258.357* (KEGG REACTION: R03008) [42]. Metabolome profiling may help elucidate the possible roles of biologically active molecules in plant control over the effectiveness of the formed symbioses.

Moreover, there was a veritable increase in expression of genes involved in flavonoid biosynthesis in roots of ‘Triumph’ and ‘Vendevil’, which may suggest the more active interaction with microsymbionts, as flavonoids have been shown to accumulate in roots during the early stages of both nodulation and mycorrhization, playing a role in molecular dialogue between plant and microorganisms and enabling symbiotic specificity. Our previous work suggested the high expression level of flavonoid biosynthesis genes in roots to be a transcriptional biomarker of pea cultivars with high EIBSM [43]; the results of the present study corroborate that idea (intriguingly, neither ‘Vendevil’ nor ‘Triumph’ were included in the set of pea cultivars used by Afonin et al. in [43]). It is known that domestication resulted in a reduction in secondary metabolites content in several legumes [44]; possibly, the content and diversity of flavonoids in roots (and root exudates) of pea may be connected with the symbiotic responsivity. Similarly, the ‘mycorrhizal dependency’, i.e., the benefit for a plant from arbuscular mycorrhizal colonization is lower in cultivated plant species than in wild species [45]. In this regard, it seems relevant to study the root flavonoid content in wild and cultivated pea varieties paying attention to the symbiotic properties of these varieties. 

Our approach combining genomic and transcriptomic sequencing allowed us to reveal biomarkers of the EIBSM, i.e., the genes that increase expression in response to inoculation in ‘Triumph’ and ‘Vendevil’, as opposed to ‘Classic’. Homologs of some genes from this group were described as mycorrhiza-specific signatures in *M. truncatula*, thus the increased expression of these genes indicates the proper response to inoculation with AM fungi. We did not find a clear response to nodule bacteria, though, probably due to contamination of the control samples. Among the signatures associated with mycorrhization, genes with the assigned function in the biosynthesis of brassinosteroids were detected; it is known that these phytohormones affect both AM and RN symbioses, playing opposite roles to gibberellins in their regulation [41,46,47]. Perhaps the fine-tuning of the balance between the development of the two symbioses represents the molecular genetic base of the EIBSM in pea. 

However, transcription markers are difficult to apply in breeding programs, while DNA-based markers are considered more suitable for the widespread use in breeding. Among the genes whose expression patterns distinguished ‘Triumph’ and ‘Vendevil’ from ‘Classic’, we were able to find candidate genes with differences in the sequence of the coding or promoter part. One of them encodes the 12-oxophytodienoate reductase 1 protein (the homolog of *M. truncatula MTR_5g006740*), which participates in the biosynthesis of the precursor of jasmonic acid [34,48]. The other, the most promising marker of EIBSM, is the gene we named *PsGLP2*, which carries the 9-bp insertion in the promoter region in ‘Vendevil’ and ‘Triumph’ and is up-regulated in response to inoculation, unlike that of ‘Classic’. The ortholog of *PsGLP2* in *M. truncatula* is *Medtr2g031270* encoding the germin-like protein (GLP) with putative function of the rhicadhesin receptor. The germin-like proteins are part of the biochemically diverse cupin superfamily that has a conserved tertiary structure with limited similarity in primary sequence [49,50]. GLPs participate in various development processes in plants, usually exhibiting enzymatic activity of oxalate oxidase and superoxide dismutase [50,51]. It has been shown that these proteins are involved in interaction with microorganisms, in particular, the establishment of symbiosis with rhizobia and AM fungi [51,52]. In this regard, the rhicadhesin receptor plays an important role in the early stages of root nodule symbiosis, as it enables the initial attachment of the bacteria to the root surface via the Ca^2+^-dependent rhicadhesin protein; without this attachment, further stages of symbiosis are impossible [51,53]. However, this is at odds with our data on nodulation, according to which ‘Classic’ formed more nodules than ‘Triumph’ or ‘Vendevil’, so *PsGLP2* probably plays another role, perhaps, associated with mycorrhiza; at least three different AM-induced GLPs seem to be involved in AM symbiosis, as shown in several studies on *M. truncatula* [49,50,52], and the closest homolog from *M. truncatula* expresses during AM formation (see Supplementary Figure S9). Also worth mentioning, despite the fact that the primary sequence is of little relevance for GLPs, *PsGLP2* is quite similar to *PsGER2a* and *PsGER2b*, both expressed in pea roots, though with unknown function [51].

Regardless of the possible function of the *PsGLP2*, this gene contains the cis-regulator of its expression (located upstream the ORF) and therefore can be easily converted into the DNA marker suitable for marker-assisted breeding. The expression of other transcriptional biomarkers identified in the present study, apparently, is regulated by trans-regulators (i.e., the sequences located far from the ORF, which may either encode transcription factor(s) or be enhancer/silencer motifs). Probably, such is the case of the genes involved in flavonoid biosynthesis. Further work in this direction may be focused on the search for transcription factors co-expressed with the biosignatures of EIBSM, on the identification of the features in promoter regions of the differentially expressed genes, and on the test of these DNA markers on the set of pea lines differing in the symbiotic responsivity. 

In general, the direct search for genetic determinants inherited by ‘Triumph’ from ‘Vendevil’ brought only limited success, since ‘Triumph’ appeared to carry an unexpectedly large portion of the ‘Vendevil’ genome (as much as one-fourth of the genes). We anticipated that, after five backcrosses, the portion of the ‘Vendevil’ genome should decrease down to about 3% (i.e., ½ ^ 5), but apparently, the genetic material used for crosses was not linear, which caused high heterogeneity in the progeny. As a result, more than 20% of the ‘Triumph’ genome came from ‘Vendevil’. At the same time, we detected the allele-specific expression only for the genes expressed in the underground parts, i.e., where the symbioses form and function, which indicates that the breeding for the symbiotic responsivity was specific and effective. Among the genes with an allele-specific expression in roots, the promising gene *PsGLP2* was identified, which will be tested in future work for its feasibility and usability as the marker of the EIBSM trait in pea.

## 4. Materials and Methods

### 4.1. Plants Material and Growth Condition

Plant material consisted of two pea (*Pisum sativum* L.) cultivars, ‘Classic’ (The Netherlands) and ‘Vendevil’ (France; = k-8274 in VIR catalog), as well as breeding line ‘Triumph’ obtained by the five backcrosses of the aforementioned cultivars [18,19].

#### Experimental Setup for the Pot Experiment

The seeds of studied genotypes were surface-sterilized by concentrated sulfuric acid for 5 min and rinsed by sterile water 5 times. Seeds were grown on sterile 1% agar medium for 5 days at 24 °C. Then, pea sprouts were planted in 5 L pots (5 plants per pot) filled with quartz sand and a mixture of mineral nutrition (1 mM NH_4_NO_3_; 2 mM KH_2_PO_4_; 2 mM Ca_3_(PO_4_)_2_; 4 mM MgSO_4_; 5 mM K_2_SO_4_; 73.6 μM H_3_BO_3_; 18.3 μM MnSO_4_; 1.53 μM ZnSO_4_; 0.64 μM CuSO_4_; 0.2 μM (NH_4_)_2_MoO_4_; 0.21 μM CoCl_2_; 37.9 μM NaFe-EDTA). An amount of 500 mL of solution was added to the pot. The weight of all pots was adjusted to the same value before planting.

Half of the plants (experimental pots) were inoculated by 2 mL of water suspension of *Rhizobium leguminosarum* bv. *viciae* RCAM1026 [54] per plant (10^7^ CFU mL^−1^) and by the AM fungus *Rhizophagus irregularis* strain BEG144 initially provided by the International Bank for the Glomeromycota (Dijon, France) and propagated in ARRIAM (St. Petersburg, Russia). *Plecthrantus australis (Lamiaceae)* was used as a host plant for *R. irregularis* cultivation. Fresh roots of *P. australis* colonized by *R. irregularis* were surface-washed with distilled water, cut into 0.5–1 cm segments and used as AM fungal inoculum (0.2 g of inoculum per plant into wells made in the sand before planting). Another half of plants (control pots) were not inoculated with *Rh. leguminosarum* bv. *viciae* RCAM1026 and AM fungi, and were only supplemented with 2 mL of water per plant before planting. The experiment was performed in a completely randomized design. 

The plants were grown in a vegetation house of the All-Russia Research Institute for Agricultural Microbiology (ARRIAM), St. Petersburg, during summer 2018 under non-controlled temperature, humidity and light conditions. Part of the pots was harvested after 4 weeks of planting (three pots for each variant, three plants from one pot were collected together and considered as one replicate). Shoots and roots were collected separately in different falcon tubes. All plants were immediately frozen in liquid nitrogen and stored at −80 °C before processing. The remaining two plants from each pot (6 plants per variant in total) were weighed on the analytical balances (OHAUS PX85, Parsippany, NJ, USA). The mass of shoots and roots was assessed separately. Nodules of these plants were also counted and weighed.

The rest of the plants were left for the growing season and harvested after 3 months, as the seeds ripened. From each variant, 22–25 plants from 5 pots were removed from sand, quickly washed by water and placed in paper bags for further drying. Then, the total weight and the seed weight were measured separately and the number of seeds was counted.

### 4.2. Statistical Analysis of Plant Growth and Yield Parameters

R ver. 4.2.3 was used to perform statistical analysis on the results of the pot experiment. Several groups were compared, including treatments (Control vs. Rh + AM) for each genotype separately and across all genotypes.

The weights of fresh shoot and root, plant and nodule biomass were examined after 4 weeks of inoculation using two-way ANOVA and Tukey’s test. Kruskal–Wallis and Dunn’s tests were used to assess the weight of one nodule and number of nodules estimated at 4 weeks after inoculation, as well as plant and seed biomass, number of seeds, and weight of 1000 seeds determined after 3 months of inoculation. Differences across groups were considered statistically significant at *p*-value < 0.05.

### 4.3. Nuclei and DNA Isolation

#### 4.3.1. Isolation of *P. sativum* Nuclei

The procedure of nuclei isolation was based on the methods presented in the Sikorskaite et al. 2013 [55] and Zerpa-Catanho et al. 2021 [56]. Nuclear DNA was isolated according to the CTAB method [57].

#### 4.3.2. Isolation of Pea Nuclear DNA

Pea nuclei dissolved in TE buffer were lysed by adding 20 μg Proteinase K (Thermo Fisher Scientific, USA) and 10 μg RNAse A (Thermo Fisher Scientific, Waltham, MA, USA), gently mixed by pipetting and incubated at 37 °C for 30 min. Then, 0.25 mL of CTAB buffer (CTAB 2%, NaCl 1.4 M, TrisCl pH 8.0 0.1 M, EDTA 20 mM) was added, mixed gently by pipetting and incubated at 62 °C for 30 min. After that, 0.5 mL of phenol–chloroform solution (Thermo Fisher Scientific, Waltham, MA, USA) was added to the sample and gently mixed until an emulsion formed. The emulsion was centrifuged for 15 min at 13,000 RCF at 4 °C in pre-cooled Eppendorf centrifuge 5430R (Eppendorf, Hamburg, Germany). The supernatant was taken in a separate 1.5 mL tube and the purification process using phenol–chloroform mix was repeated. 

Then, 2.5 V of pre-cooled 96% ethanol and NaCl (the volume was calculated so that its final concentration in solution was 0.2 M) were added to the aqueous supernatant and gently mixed by inverting the tube. The sample was incubated at −20 °C overnight. 

The tube was centrifuged for 15 min at 16,000 RCF at 4 °C in a pre-cooled Eppendorf centrifuge 5430R (Eppendorf, Hamburg, Germany). The supernatant was carefully removed and the precipitate was gently washed in 70% ethanol (10 min) three times. After the final centrifugation the sample was dried for 5 min at room temperature, 40 μL of TE buffer (AM9849, Invitrogen, Carlsbad, USA) was added to the sample. The tube was incubated at 4 °C for a night. Then, DNA solution was mixed by pipet tip. DNA concentration was measured using the dsDNA Quantitation Broad Range Kit (Invitrogen, Carlsbad, USA) and NanoDrop OneC (Thermo Fisher Scientific, Wiltham, USA).

### 4.4. Whole Genome Sequencing and Reads Processing

Shotgun genomic libraries were prepared using the TruSeq DNA PCR-Free Kit. Libraries were sequenced using Illumina NovaSeq 6000 (Illumina, San Diego, CA, USA) in Sirius University of Science and Technology (Sirius, Russia). The resulting paired-end reads were 150 nucleotides in length. The raw reads are deposited in the NCBI SRA database under accession number PRJNA1036824.

For each genome, raw reads were processed to cut off adaptor sequences and remove low-quality sequences using BBDuk from the BBMap package (https://sourceforge.net/projects/bbmap/ (accessed on 12 December 2023)) [58]. High-quality reads were mapped to the reference *P. sativum* cv. ‘Frisson’ genome assembly (NCBI accession number: JANEYU000000000) using Bowtie2 (ver. 2.3.5.1) [59]. In total, more than 90% reads were mapped to the reference. 

### 4.5. SNV Calling and Identification of Genome Regions Inherited by ‘Triumph’ from ‘Vendevil’

Sam and bam format files were processed using Samtools ver. 1.10 and Bcftools ver. 1.10.2 [60] was used to call variants and filter out low-quality variants. Annotation of obtained variants was conducted in SnpEff ver. 5.2 [61]. Then, variants located within the protein-coding genes were retained. 

In order to identify the portion of the genome of ‘Triumph’ inherited from ‘Vendevil’, variants were found that are common to both genotypes and differ from ‘Classic’. Association of the assembled contigs with chromosomes was conducted based on homology with the *M. truncatula* jemalong A17 genome assembly.

### 4.6. Functional Analysis of Genes

Functional annotation of *P. sativum* genes was obtained using Mercator4 framework ver. 6.0 [62] and by homology with the *M. truncatula* Jemalong A17 genome. Trinotate suite ver. 4.0.2 [63] was used to obtain GO terms for pea genes. GO enrichment analysis was conducted in topGO package ver. 2.54.0 (https://bioconductor.org/packages/release/bioc/html/topGO.html (accessed on 12 December 2023)) [64] using the weight01 algorithm and Fisher’s exact test and further visualization was performed in ggplot2 package ver. 3.4.4 [65].

### 4.7. RNA Isolation

The roots were ground using mortar and pestle in liquid nitrogen, and RNA was isolated using Trizol (Thermo Fisher Scientific, Waltham, MA, USA) according to the manufacturer’s protocol with minor changes. The RNA quality was assessed using gel electrophoresis in 1% agarose gel, the RNA concentration was measured using a Qubit Fluorometer and Qubit RNA BR Assay Kit (Thermo Fisher Scientific). The 3′ MACE sequencing libraries were prepared from RNA samples using a 3′ MACE kit (GenXPro GmbH, Frankfurt am Main, Germany) and sequenced on Illumina HiSeq X (Illumina, San Diego, CA, USA) at Macrogen (Seoul, Republic of Korea). The raw data were deposited in the NCBI SRA database under accession number PRJNA1036824.

### 4.8. Transcriptome Reads Processing

All raw reads were processed using BBDuk to quality and adapter trimming. Clean reads were mapped to the reference genome of pea cv. ‘Frisson’ (NCBI accession number: JANEYU000000000) using STAR ver. 2.7.11 [66]. The quantification stage was conducted in featureCounts from Subread package ver. 2.0.6 [67].

### 4.9. Differential Expression Analysis

Differential expression analysis was performed using DESeq2 ver. 1.40.2 package [68] in R programming environment ver. 4.3. The differentially expressed genes were considered to be significant at the level of the adjusted *p*-value of < 0.05.

The heatmap showing gene expression patterns was based on a 1-Pearson correlation matrix calculated on normalized per million and logarithmic (log2) expression values transformed into a z-score (which gives the number of standard deviations that a value is away from the mean of all the values in the same gene) using edgeR ver. 3.20.9 [69] and heatmap function in R. The expression values of the three biological replicates for a particular group were averaged. All genes with low expression values (less than 10 TPMs across all samples) were removed.

### 4.10. Phylogenetic Analysis

Phylogenetic analysis was conducted based on alignment of protein sequences of the genes encoding germin-like and rhicadhesin receptor proteins of *M. truncatula* and *P. sativum*. Amino acid sequences were aligned using MAFFT ver. 7.453 [70], and a phylogenetic tree was constructed in ape ver. 5.7-1 [71] and visualized in GGTREE R package ver. 1.10.5 [72].

### 4.11. Real-Time qRT-PCR

Three independent biological replicates of control and double-inoculated root tissue were used for RNA extraction for qRT-PCR assays. The reverse transcription reactions were carried out by ThermoScientific RevertAid First Strand cDNA Syntesis kit (Thermo Fisher Scientific). Designed transcript-specific primers are presented in Table S6. All reactions were performed on an CFX96 Touch Real-Time PCR Detection System (Bio-Rad, Hercules, CA, USA) with three technical replicates. Each reaction was performed in a total volume of 10 μL including 0.3 μM primer pairs, 1 μL diluted cDNA, and 2 μL 5× SYBR Green qPCRmix-HS SYBR (Evrogen, Moscow, Russia). The amplification reactions were incubated at 95 °C for 30 s, followed by 39 cycles of 95 °C for 5 s, 58 °C for 30 s, and 7 °C for 30 s. *P. sativum GapC1* gene was used as a reference gene. Relative gene expression levels were calculated using the 2^−∆∆*C*t^ method. Statistical analysis of the results was performed on 2deltaCT values using ANOVA. Pearson correlation coefficient was calculated using default R function “corr”.

## Figures and Tables

**Table 1:** Quantitative distribution of genes inherited by ‘Triumph’ from ‘Vendevil’ by chromosomes/linkage groups of *P. sativum*.

Chromosome/Linkage Group	Number of Genes with Variations	% of All Genes on a Chromosome
Chr2LGI	1252	37%
Chr6LGII	1093	35%
Chr5LGIII	393	9%
Chr4LGIV	232	6%
Chr3LGV	331	6%
Chr1LGVI	193	3%
Chr7LGVII	509	6%

**Table 2:** Differential up-regulated genes involved in hormonal regulation in response to double inoculation that are common to ‘Triumph’ and ‘Vendevil’.

*P. sativum* Gene ID	*M. truncatula* Homologous Gene ID	Annotation	Biological Process
*evm.TU.contig_464.240*	*MTR_6g034940*	Abscisic acid 8-hydroxylase 3	ABA catabolism
*evm.TU.scaffold_2084.189*	*MTR_7g102460*	UDP-glycosyltransferase 73C1	cytokinin catabolism
*evm.TU.scaffold_2084.193*	*MTR_7g102460*	UDP-glycosyltransferase 73C1	cytokinin catabolism
*evm.TU.scaffold_4814.82*	*MTR_5g082520*	steroid 3-dehydrogenase (CPD)	BR biosynthesis

**Table 3:** Differential up-regulated genes related to biosynthetic and metabolic pathways of biologically active molecules that are shared by ‘Triumph’ and ‘Vendevil’ in response to double inoculation.

*P. sativum* Gene ID	*M. truncatula* Homologous Gene ID	Encoded Enzyme	Biological Process
*evm.TU.contig_1755.59*	*MTR_5g033510*	tryptophan aminotransferase	auxin biosynthesis
*evm.TU.scaffold_258.357*	*MTR_1g034120*	2-oxoglutarate-dependent dioxygenase	gibberellin biosynthesis and metabolism
*evm.TU.contig_363.85*	*MTR_2g098490*	4-coumarate-CoA ligase-like 9	phenylpropanoid pathway
*evm.TU.scaffold_1663.185*	*MTR_3g021440*	Isoliquiritigenin 2’-O-methyltransferase	flavonoid biosynthesis
*evm.TU.scaffold_341.76*	*MTR_0536s001*	UDP-glucose flavonoid 3-O-glucosyltransferase	flavonoid biosynthesis

## Data Availability

The DNA and RNA sequencing data have been uploaded to the NCBI under BioProject accession number PRJNA1036824.

## Supplementary Materials

The following supporting information can be downloaded at https://www.mdpi.com/article/10.3390/plants13010078/s1. Figure S1: Variants distribution across genomic regions; Figure S2: Stages of variants filtering; Figure S3: GO enrichment analysis of genes with indels that are common for ‘Triumph’ and ‘Vendevil’; Figure S4: Nodule number and nodule weight across genotypes; Figure S5: Fresh weight of different plant’s parts across genotypes; Figure S6: Shoot weight, seed weight and seed number across genotypes; Figure S7: PCA analysis of transcriptomic data; Figure S8: Venn diagram demonstrating transcriptomic differences between ‘Triumph’ and ‘Vendevil’ compared to ‘Classic’; Figure S9: Expression analysis of *M. truncatula*’s homolog of *PsGLP2* obtained using MtGEA; Figure S10: A phylogenetic tree constructed based on the alignment of the sequence of genes encoding germin-like proteins and rhicadhesin receptors in *P. sativum* and *M. truncatula*; Figure S11: Results of qRT-PCR analysis and comparison with transcriptomic data; Table S1: Reads processing description; Table S2: Common up-regulated DEGs in ‘Classic’, ‘Triumph’ and ‘Vendevil’; Table S3: Down-regulated DEGs in response to double inoculation; Table S4: Common up-regulated DEGs in response to double inoculation in ‘Triumph’ and ‘Vendevil’; Table S5: Common DEGs in ‘Triumph’ and ‘Vendevil’ compared to ‘Classic’; Table S6: List of primers for qRT-PCR validation of transcriptomic data.

## References

[1] Wang D., Dong W., Murray J., Wang E. (2022). Innovation and Appropriation in Mycorrhizal and Rhizobial Symbioses. Plant Cell.

[2] Dilworth M.J., James E.K., Sprent J.I., Newton W.E. (2008). Nitrogen-Fixing Leguminous Symbioses.

[3] Liu A., Ku Y.S., Contador C.A., Lam H.M. (2020). The Impacts of Domestication and Agricultural Practices on Legume Nutrient Acquisition through Symbiosis with Rhizobia and Arbuscular Mycorrhizal Fungi. Front. Genet..

[4] Peoples M.B., Herridge D.F., Ladha J.K. (1995). Biological Nitrogen Fixation: An Efficient Source of Nitrogen for Sustainable Agricultural Production?. Plant Soil.

[5] Zahran H.H. (1999). Rhizobium-Legume Symbiosis and Nitrogen Fixation under Severe Conditions and in an Arid Climate. Microbiol. Mol. Biol. Rev..

[6] Clúa J., Roda C., Zanetti M.E., Blanco F.A. (2018). Compatibility between Legumes and Rhizobia for the Establishment of a Successful Nitrogen-Fixing Symbiosis. Genes.

[7] Fall A.F., Nakabonge G., Ssekandi J., Founoune-Mboup H., Apori S.O., Ndiaye A., Badji A., Ngom K. (2022). Roles of Arbuscular Mycorrhizal Fungi on Soil Fertility: Contribution in the Improvement of Physical, Chemical, and Biological Properties of the Soil. Front. Fungal Biol..

[8] Shtark O.Y., Zhukov V.A., Sulima A.S., Singh R., Naumkina T.S., Borisov A.Y. (2015). Prospects for the Use of Multi-Component Symbiotic Systems of the Legumes. Ecol. Genet..

[9] Roy S., Liu W., Nandety R.S., Crook A., Mysore K.S., Pislariu C.I., Frugoli J., Dickstein R., Udvardi M.K. (2020). Celebrating 20 Years of Genetic Discoveries in Legume Nodulation and Symbiotic Nitrogen Fixation. Plant Cell.

[10] Bourion V., Heulin-Gotty K., Aubert V., Tisseyre P., Chabert-Martinello M., Pervent M., Delaitre C., Vile D., Siol M., Duc G. (2018). Co-Inoculation of a Pea Core-Collection with Diverse Rhizobial Strains Shows Competitiveness for Nodulation and Efficiency of Nitrogen Fixation Are Distinct Traits in the Interaction. Front. Plant Sci..

[11] Lindström K., Mousavi S.A. (2020). Effectiveness of Nitrogen Fixation in Rhizobia. Microb. Biotechnol..

[12] Van Der Heijden M.G.A. (2002). Arbuscular Mycorrhizal Fungi as a Determinant of Plant Diversity: In Search of Underlying Mechanisms and General Principles. Mycorrhizal Ecology.

[13] Pandey A.K., Rubiales D., Wang Y., Fang P., Sun T., Liu N., Xu P. (2021). Omics Resources and Omics-Enabled Approaches for Achieving High Productivity and Improved Quality in Pea (*Pisum Sativum* L.). Theor. Appl. Genet..

[14] Bagheri M., Santos C.S., Rubiales D., Vasconcelos M.W. (2023). Challenges in Pea Breeding for Tolerance to Drought: Status and Prospects. Ann. Appl. Biol..

[15] Rubiales D., Barilli E., Rispail N. (2023). Breeding for Biotic Stress Resistance in Pea. Agriculture.

[16] Shtark O.Y., Borisov A.Y., Zhukov V.A., Tikhonovich I.A. (2012). Mutually Beneficial Legume Symbioses with Soil Microbes and Their Potential for Plant Production. Symbiosis.

[17] Yakobi L.M., Kukalev A.S., Ushakov K.V., Tsyganov V.E., Naumkina T.S., Provorov N.A., Borisov A.Y., Tikhonovich I.A. (2000). Polymorphism of Garden Pea Forms by the Effectiveness of Symbiosis with the Endomycorrhizal Fungus *Glomus Sp*. under Conditions of Inoculation with Rhizobia. Agric. Biol. Sel’skokhozyaistvennaya Biol..

[18] Zhukov V.A., Zhernakov A.I., Sulima A.S., Kulaeva O.A., Kliukova M.S., Afonin A.M., Shtark O.Y., Tikhonovich I.A. (2021). Association Study of Symbiotic Genes in Pea (*Pisum Sativum* L.) Cultivars Grown in Symbiotic Conditions. Agronomy.

[19] Naumkina T.S. (2007). Breeding Peas (*Pisum Sativum* L.) to Increase the Efficiency of Symbiotic Nitrogen Fixation, Diss. Dr. Sci. State Scientific Institution All-Russian Research Institute of Grain Legumes and Cereals of the Russian Agricultural Academy. https://www.dissercat.com/content/selektsiya-gorokha-pisum-sativum-l-na-povyshenie-effektivnosti-simbioticheskoi-azotfiksatsii/read.

[20] Shtark O.Y., Danilova T.N., Naumkina T.S., Vasilchikov A.G., Chebotar V.K., Kazakov A.E., Zhernakov A.I., Nemankin T.A., Prilepskaya N.A., Borisov A.U. (2006). Analysis of Pea (*Pisum Sativum* L.) Source Material for Breeding of Cultivars with High Symbiotic Potential and Choice of Criteria for Its Evaluation. Ecol. Genet..

[21] Zorin E.A., Kliukova M.S., Afonin A.M., Gribchenko E.S., Gordon M.L., Sulima A.S., Zhernakov A.I., Kulaeva O.A., Romanyuk D.A., Kusakin P.G. (2022). A Variable Gene Family Encoding Nodule-Specific Cysteine-Rich Peptides in Pea (*Pisum Sativum* L.). Front. Plant Sci..

[22] Kaiser B.N., Moreau S., Castelli J., Thomson R., Lambert A., Bogliolo S., Puppo A., Day D.A. (2003). The Soybean NRAMP Homologue, GmDMT1, Is a Symbiotic Divalent Metal Transporter Capable of Ferrous Iron Transport. Plant J..

[23] Middleton P.H., Jakab J., Penmetsa R.V., Starker C.G., Doll J., Kalo P., Prabhu R., Marsh J.F., Mitra R.M., Kereszt A. (2007). An ERF Transcription Factor in *Medicago truncatula* That Is Essential for Nod Factor Signal Transduction. Plant Cell Online.

[24] Wu F.-Q., Zhang X.-M., Li D.-M., Fu Y.-F. (2011). Ectopic Expression Reveals a Conserved PHYB Homolog in Soybean. PLoS ONE.

[25] Haney C.H., Riely B.K., Tricoli D.M., Cook D.R., Ehrhardt D.W., Long S.R. (2011). Symbiotic Rhizobia Bacteria Trigger a Change in Localization and Dynamics of the *Medicago truncatula* Receptor Kinase LYK3. Plant Cell.

[26] Mbengue M., Camut S., de Carvalho-Niebel F., Deslandes L., Froidure S., Klaus-Heisen D., Moreau S., Rivas S., Timmers T., Hervé C. (2010). The *Medicago truncatula* E3 Ubiquitin Ligase PUB1 Interacts with the LYK3 Symbiotic Receptor and Negatively Regulates Infection and Nodulation. Plant Cell.

[27] Jia H., Ren H., Gu M., Zhao J., Sun S., Zhang X., Chen J., Wu P., Xu G. (2011). The Phosphate Transporter Gene Ospht1;8 Is Involved in Phosphate Homeostasis in Rice. Plant Physiol..

[28] Breuillin-Sessoms F., Floss D.S., Karen Gomez S., Pumplin N., Ding Y., Levesque-Tremblay V., Noar R.D., Daniels D.A., Bravo A., Eaglesham J.B. (2015). Suppression of Arbuscule Degeneration in *Medicago truncatula* Phosphate Transporter4 Mutants Is Dependent on the Ammonium Transporter 2 Family Protein AMT2;3. Plant Cell.

[29] Gutjahr C., Radovanovic D., Geoffroy J., Zhang Q., Siegler H., Chiapello M., Casieri L., An K., An G., Guiderdoni E. (2012). The Half-size ABC Transporters STR1 and STR2 Are Indispensable for Mycorrhizal Arbuscule Formation in Rice. Plant J..

[30] Zhang Q., Blaylock L.A., Harrison M.J. (2010). Two Medicago Truncatula Half-ABC Transporters Are Essential for Arbuscule Development in Arbuscular Mycorrhizal Symbiosis. Plant Cell.

[31] Wallace I.S., Choi W.G., Roberts D.M. (2006). The Structure, Function and Regulation of the Nodulin 26-like Intrinsic Protein Family of Plant Aquaglyceroporins. Biochim. Biophys. Acta Biomembr..

[32] Parker J.L., Newstead S. (2014). Molecular Basis of Nitrate Uptake by the Plant Nitrate Transporter NRT1.1. Nature.

[33] Mohammadi-Dehcheshmeh M., Niazi A., Ebrahimi M., Tahsili M., Nurollah Z., Ebrahimi Khaksefid R., Ebrahimi M., Ebrahimie E. (2018). Unified Transcriptomic Signature of Arbuscular Mycorrhiza Colonization in Roots of *Medicago truncatula* by Integration of Machine Learning, Promoter Analysis, and Direct Merging Meta-Analysis. Front. Plant Sci..

[34] Schaller F., Biesgen C., Müssig C., Altmann T., Weiler E.W. (2000). 12-Oxophytodienoate Reductase 3 (OPR3) Is the Isoenzyme Involved in Jasmonate Biosynthesis. Planta.

[35] Goyal R.K., Mattoo A.K., Schmidt M.A. (2021). Rhizobial–Host Interactions and Symbiotic Nitrogen Fixation in Legume Crops Toward Agriculture Sustainability. Front. Microbiol..

[36] Sinjushin A., Semenova E., Vishnyakova M. (2022). Usage of Morphological Mutations for Improvement of a Garden Pea (*Pisum sativum*): The Experience of Breeding in Russia. Agronomy.

[37] Kuzmicheva Y.V., Shaposhnikov A.I., Azarova T.S., Petrova S.N., Naumkina T.S., Borisov A.Y., Belimov A.A., Kravchenko L.V., Parakhin N.V., Tikhonovich I.A. (2014). Composition of Root Exometabolites of the Symbiotically Effective Pea Cultivar Triumph and Its Parental Forms. Russ. J. Plant Physiol..

[38] Hedden P., Thomas S.G. (2012). Gibberellin Biosynthesis and Its Regulation. Biochem. J..

[39] Lange T., Lange M.J.P. (2020). The Multifunctional Dioxygenases of Gibberellin Synthesis. Plant Cell Physiol..

[40] Kawai Y., Ono E., Mizutani M. (2014). Evolution and Diversity of the 2-Oxoglutarate-Dependent Dioxygenase Superfamily in Plants. Plant J..

[41] McAdam E.L., Reid J.B., Foo E. (2018). Gibberellins Promote Nodule Organogenesis but Inhibit the Infection Stages of Nodulation. J. Exp. Bot..

[42] Igielski R., Kępczyńska E. (2017). Gene Expression and Metabolite Profiling of Gibberellin Biosynthesis during Induction of Somatic Embryogenesis in *Medicago truncatula* Gaertn. PLoS ONE.

[43] Afonin A.M., Gribchenko E.S., Zorin E.A., Sulima A.S., Romanyuk D.A., Zhernakov A.I., Shtark O.Y., Akhtemova G.A., Zhukov V.A. (2021). Unique Transcriptome Features of Pea (*Pisum sativum* L.) Lines with Differing Responses to Beneficial Soil Microorganisms. Ecol. Genet..

[44] Ku Y.S., Contador C.A., Ng M.S., Yu J., Chung G., Lam H.M. (2020). The Effects of Domestication on Secondary Metabolite Composition in Legumes. Front. Genet..

[45] Tawaraya K. (2003). Arbuscular Mycorrhizal Dependency of Different Plant Species and Cultivars. Soil Sci. Plant Nutr..

[46] McGuiness P.N., Reid J.B., Foo E. (2019). The Role of Gibberellins and Brassinosteroids in Nodulation and Arbuscular Mycorrhizal Associations. Front. Plant Sci..

[47] Foo E., Ross J.J., Jones W.T., Reid J.B. (2013). Plant Hormones in Arbuscular Mycorrhizal Symbioses: An Emerging Role for Gibberellins. Ann. Bot..

[48] Schaller F. (2001). Enzymes of the Biosynthesis of Octadecanoid-derived Signalling Molecules. J. Exp. Bot..

[49] Dunwell J.M., Gibbings J.G., Mahmood T., Saqlan Naqvi S.M. (2008). Germin and Germin-like Proteins: Evolution, Structure, and Function. CRC. Crit. Rev. Plant Sci..

[50] Barman A.R., Banerjee J. (2015). Versatility of Germin-like Proteins in Their Sequences, Expressions, and Functions. Funct. Integr. Genomics.

[51] Gucciardo S., Wisniewski J.P., Brewin N.J., Bornemann S. (2007). A Germin-like Protein with Superoxide Dismutase Activity in Pea Nodules with High Protein Sequence Identity to a Putative Rhicadhesin Receptor. J. Exp. Bot..

[52] Doll J., Hause B., Demchenko K., Pawlowski K., Krajinski F. (2003). A Member of the Germin-Like Protein Family Is a Highly Conserved Mycorrhiza-Specific Induced Gene. Plant Cell Physiol..

[53] Chakraborty S., Driscoll H.E., Abrahante J.E., Zhang F., Fisher R.F., Harris J.M. (2021). Salt Stress Enhances Early Symbiotic Gene Expression in *Medicago truncatula* and Induces a Stress-Specific Set of *Rhizobium*-Responsive Genes. Mol. Plant-Microbe Interact..

[54] Afonin A., Sulima A., Zhernakov A., Zhukov V. (2017). Draft Genome of the Strain RCAM1026 *Rhizobium leguminosarum* Bv. Viciae. Genomics Data.

[55] Sikorskaite S., Rajamäki M.L., Baniulis D., Stanys V., Valkonen J.P.T. (2013). Protocol: Optimised Methodology for Isolation of Nuclei from Leaves of Species in the Solanaceae and Rosaceae Families. Plant Methods.

[56] Zerpa-Catanho D., Zhang X., Song J., Hernandez A.G., Ming R. (2021). Ultra-Long DNA Molecule Isolation from Plant Nuclei for Ultra-Long Read Genome Sequencing. STAR Protoc..

[57] Rogers S.O., Bendich A.J. (1985). Extraction of DNA from Milligram Amounts of Fresh, Herbarium and Mummified Plant Tissues. Plant Mol. Biol..

[58] Bushnell B. (2014). BBMap: A Fast, Accurate, Splice-Aware Aligner.

[59] Langmead B., Salzberg S.L. (2012). Fast Gapped-Read Alignment with Bowtie 2. Nat. Methods.

[60] Danecek P., Bonfield J.K., Liddle J., Marshall J., Ohan V., Pollard M.O., Whitwham A., Keane T., McCarthy S.A., Davies R.M. (2021). Twelve Years of SAMtools and BCFtools. Gigascience.

[61] Cingolani P., Platts A., Wang L.L., Coon M., Nguyen T., Wang L., Land S.J., Lu X., Ruden D.M. (2012). A Program for Annotating and Predicting the Effects of Single Nucleotide Polymorphisms, SnpEff: SNPs in the Genome of *Drosophila melanogaster* Strain W1118; Iso-2; Iso-3. Fly.

[62] Bolger M., Schwacke R., Usadel B. (2021). MapMan Visualization of RNA-Seq Data Using Mercator4 Functional Annotations. Solanum tuberosum: Methods and Protocols.

[63] Griffith M., Walker J.R., Spies N.C., Ainscough B.J., Griffith O.L. (2015). Informatics for RNA Sequencing: A Web Resource for Analysis on the Cloud. PLoS Comput. Biol..

[64] Alexa A., Rahnenfuhrer J. (2023). TopGO: Enrichment Analysis for Gene Ontology. R Package Version 2023.

[65] Wickham H. (2016). Getting Started with Ggplot2. ggplot2: Elegant Graphics for Data Analysis.

[66] Dobin A., Davis C.A., Schlesinger F., Drenkow J., Zaleski C., Jha S., Batut P., Chaisson M., Gingeras T.R. (2013). STAR: Ultrafast Universal RNA-Seq Aligner. Bioinformatics.

[67] Liao Y., Smyth G.K., Shi W. (2014). FeatureCounts: An Efficient General Purpose Program for Assigning Sequence Reads to Genomic Features. Bioinformatics.

[68] Love M.I., Huber W., Anders S. (2014). Moderated Estimation of Fold Change and Dispersion for RNA-Seq Data with DESeq2. Genome Biol..

[69] Robinson M.D., McCarthy D.J., Smyth G.K. (2010). EdgeR: A Bioconductor Package for Differential Expression Analysis of Digital Gene Expression Data. Bioinformatics.

[70] Katoh K., Misawa K., Kuma K., Miyata T. (2002). MAFFT: A Novel Method for Rapid Multiple Sequence Alignment Based on Fast Fourier Transform. Nucleic Acids Res..

[71] Paradis E., Schliep K. (2019). Ape 5.0: An Environment for Modern Phylogenetics and Evolutionary Analyses in R. Bioinformatics.

[72] Yu G., Smith D.K., Zhu H., Guan Y., Lam T.T. (2017). Ggtree: An R Package for Visualization and Annotation of Phylogenetic Trees with Their Covariates and Other Associated Data. Methods Ecol. Evol..

