# Supplementary material for: Genomic and Transcriptomic Analysis of Pea (Pisum sativum L.) Breeding Line ‘Triumph’ with High Symbiotic Responsivity
Source: Plants (Basel). 2023 Dec 26;13(1):78. doi: 10.3390/plants13010078 (PMC10781049; doi:10.3390/plants13010078)
Supplement: Supplementary file 1 [file plants-13-00078-s001.zip › plants-2747820-supplementary Figures.pdf]

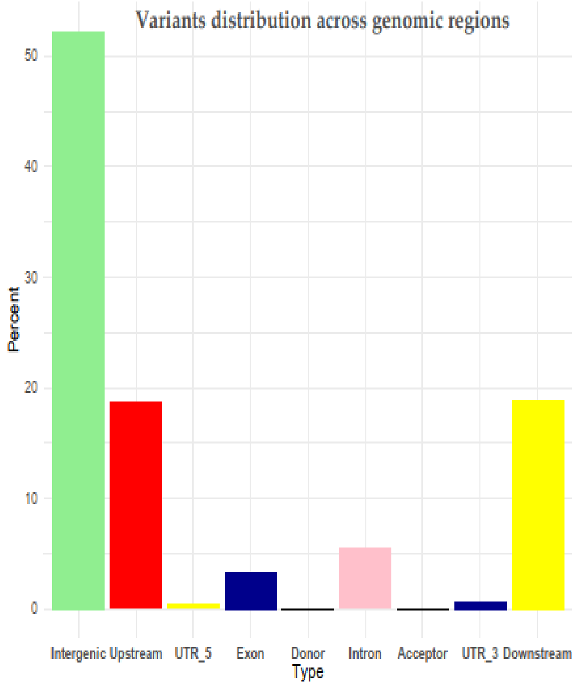

Figure S1. Variants distribution across genomic regions.

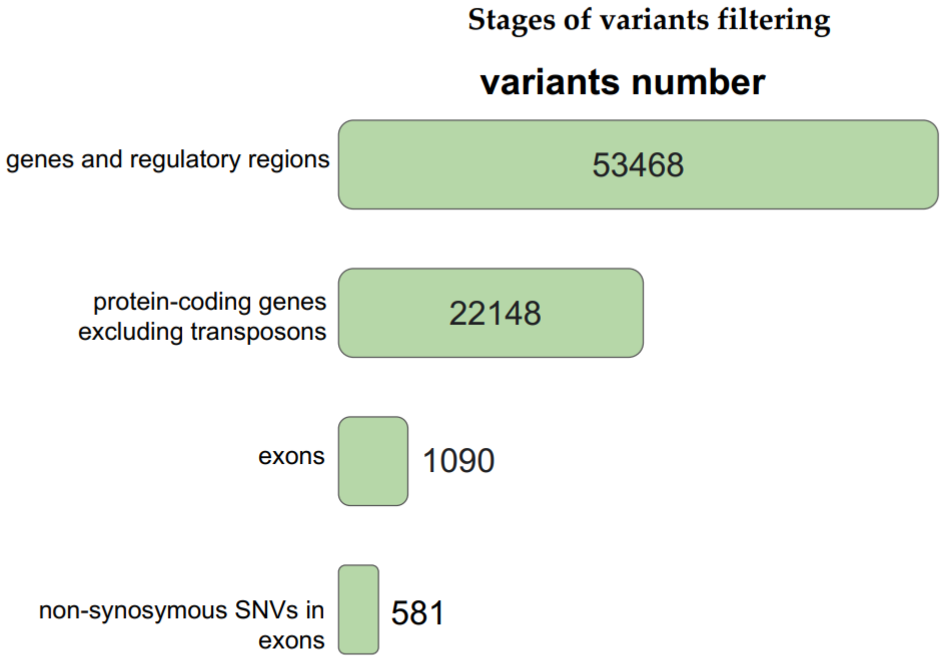

Figure S2. Stages of variants filtering.

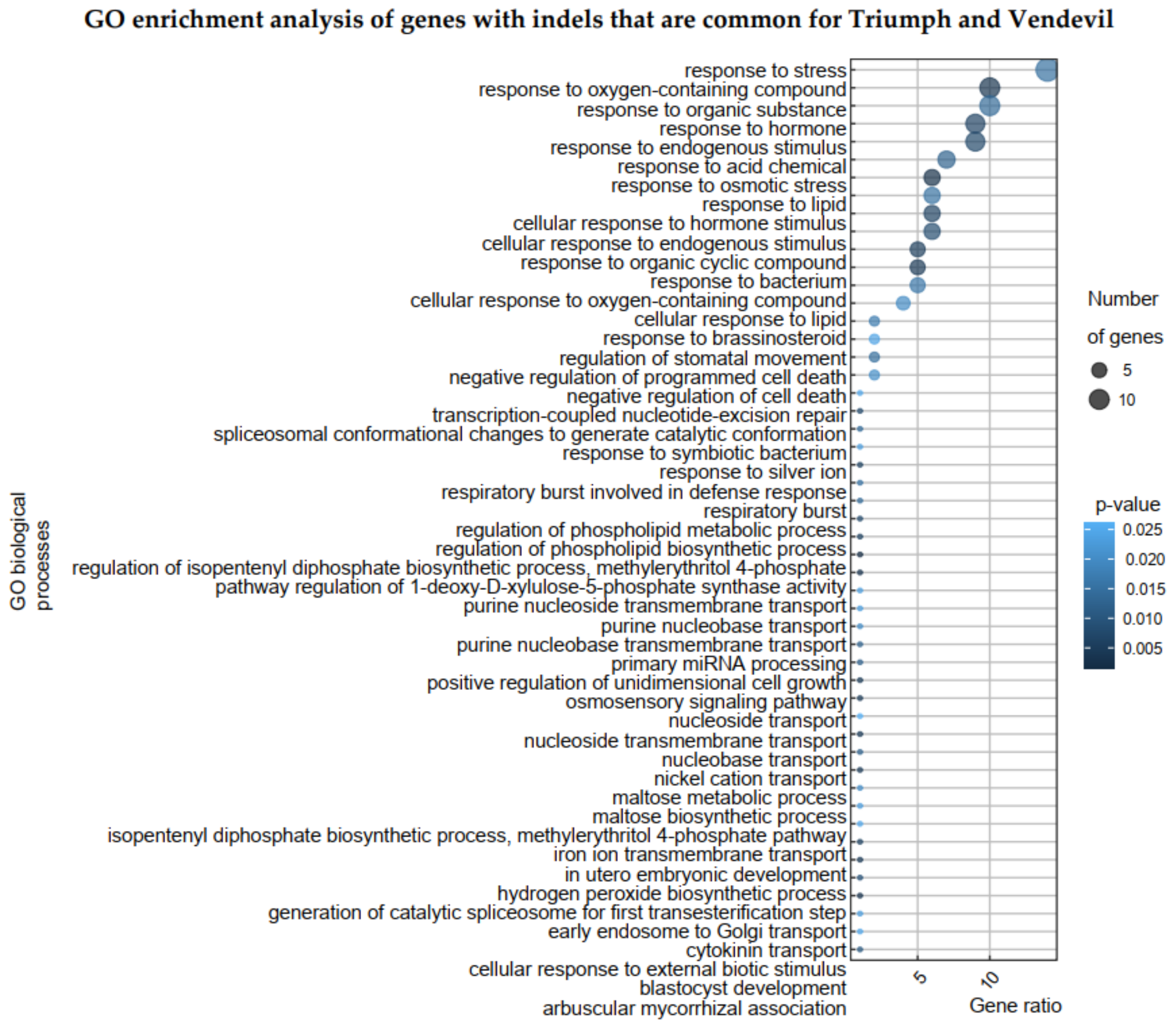

Figure S3. GO enrichment analysis of genes with indels that are common for 'Triumph' and 'Vendevil'.

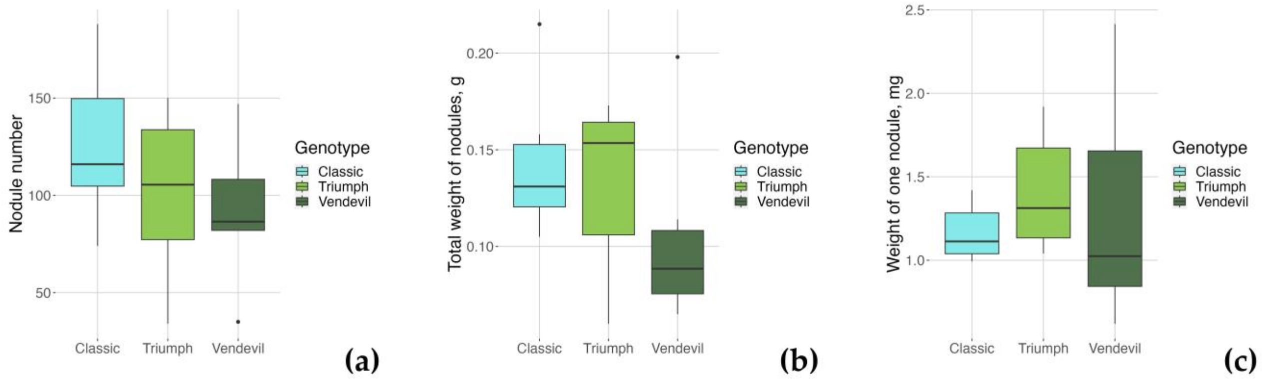

Figure S4. Nodule number and nodule weight across genotypes.

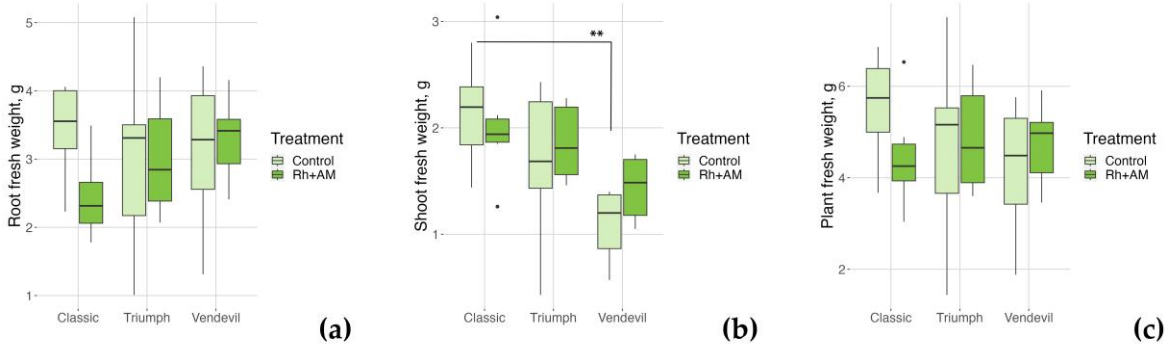

Figure S5. Fresh weight of different plant's parts across genotypes.

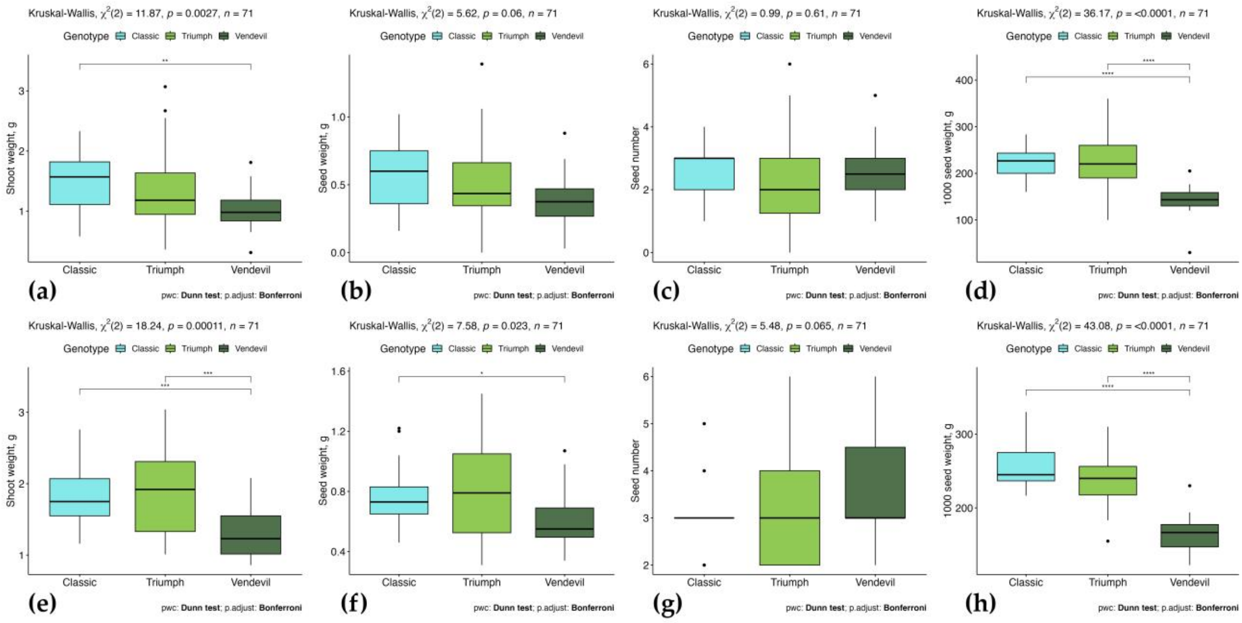

Figure S6. Shoot weight, seed weight and seed number across genotypes.

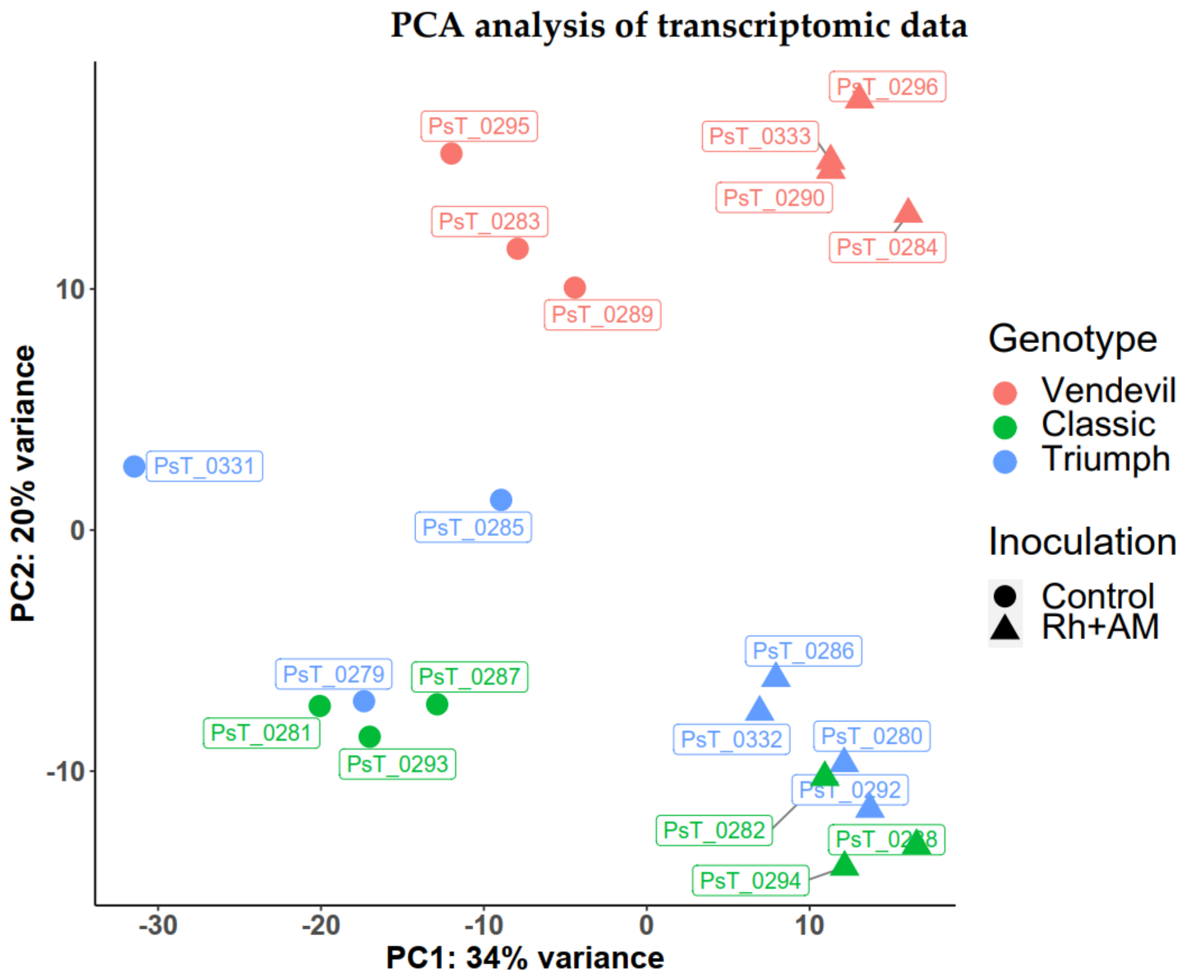

Figure S7. PCA analysis of transcriptomic data.

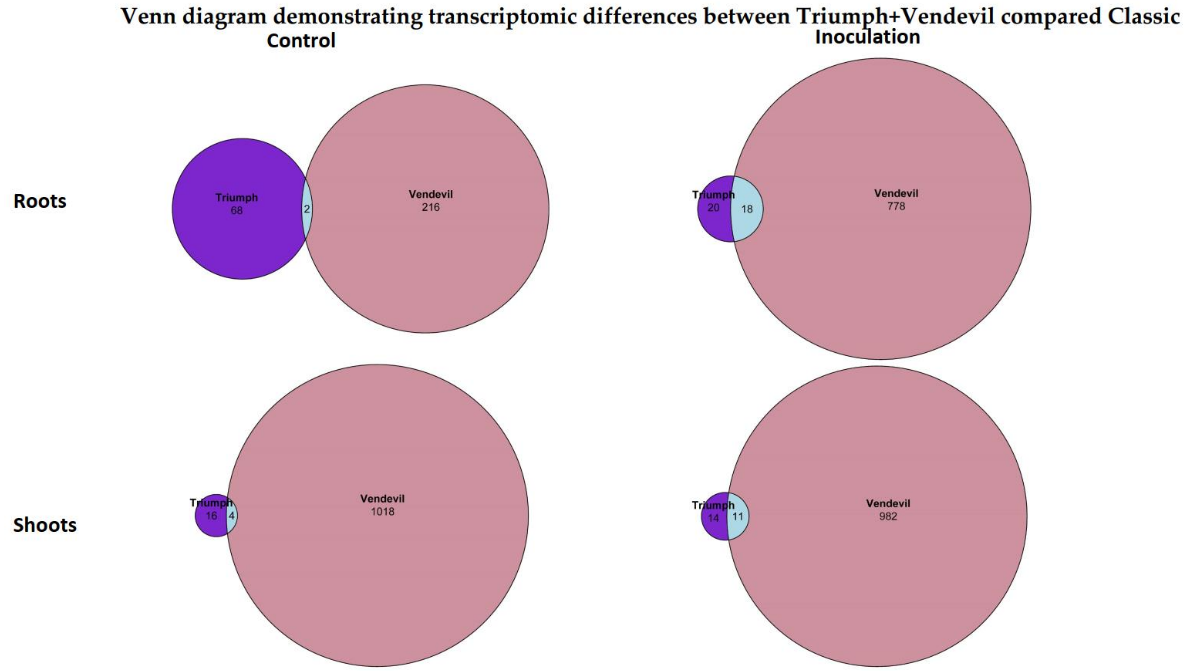

Figure S8. Venn diagram demonstrating transcriptomic differences between 'Triumph' and 'Vendevil' compared to 'Classic'.

Expression analysis of *M. truncatula's* homolog of *PsGLP2* obtained using MtGEA

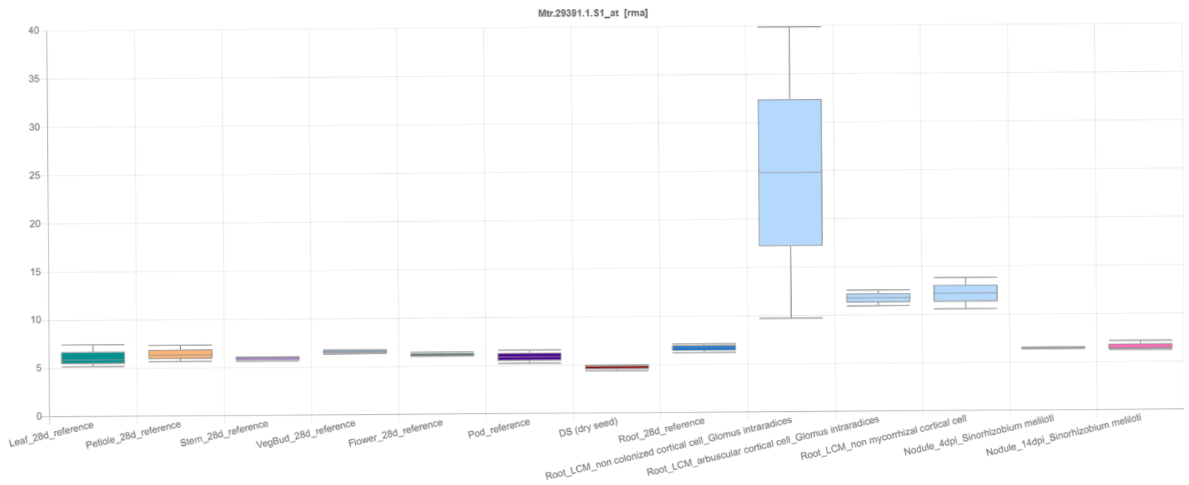

Figure S9. Expression analysis of *M. truncatula*'s homolog of *PsGLP2* obtained using MtGEA.

A phylogenetic tree constructed based on the alignment of the sequence of genes encoding germin-like proteins and rhicadhesin receptors in *P. sativum* and *M. truncatula.*

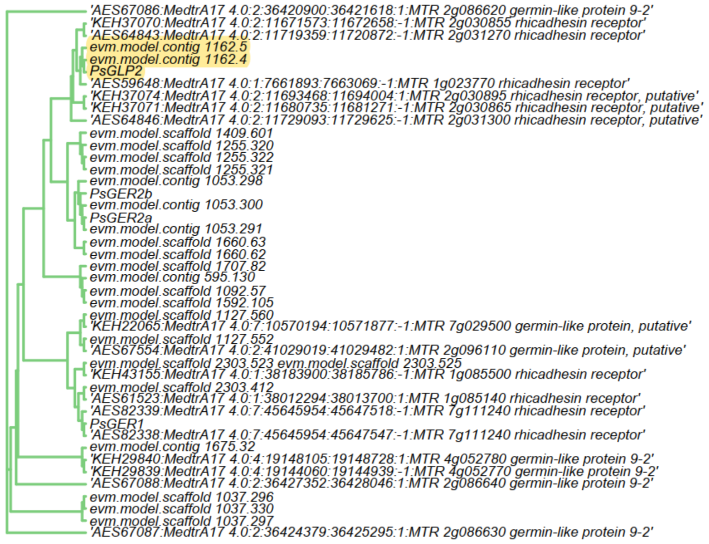

Figure S10. A phylogenetic tree constructed based on the alignment of the sequence of genes encoding germin-like proteins and rhicadhesin receptors in *P. sativum* and *M. truncatula*.

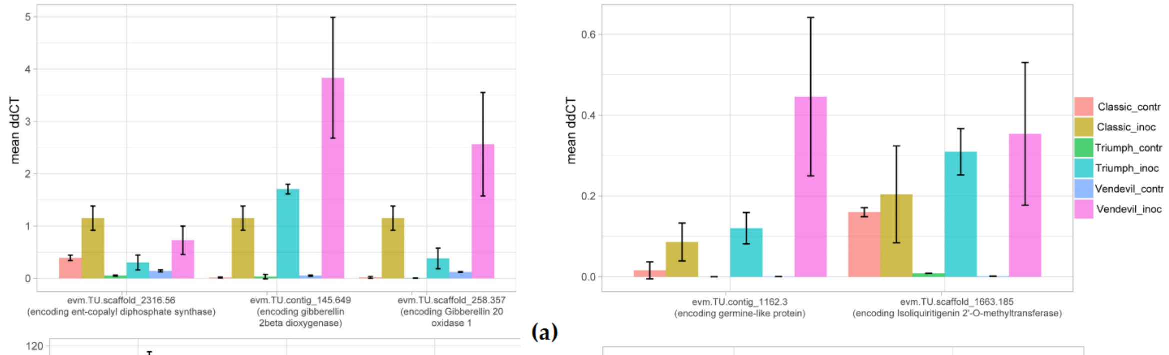

(a)

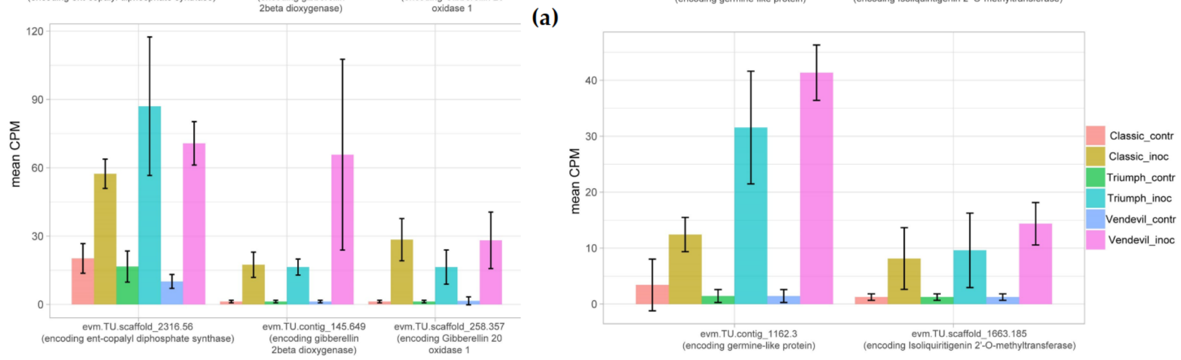

(b)

Figure S11. Results of qRT-PCR analysis and comparison with transcriptomic data.
